# Antibiotic-Resistant Gram Negative Bacilli in Meals Delivered at a General Hospital, Italy

**DOI:** 10.1155/2009/476150

**Published:** 2009-09-10

**Authors:** Maria Rosa Anna Plano, Anna Maria Di Noto, Alberto Firenze, Sonia Sciortino, Caterina Mammina

**Affiliations:** ^1^Department of Sciences for Health Promotion “G. D’Alessandro,” University of Palermo, 90127 Palermo, Italy; ^2^Istituto Zooprofilattico Sperimentale della Sicilia “A. Mirri,” Palermo, Italy

## Abstract

This study aimed at detecting the presence of antibiotic-resistant Gram-negatives in samples of meals delivered at the University General Hospital of Palermo, Italy. Antibiotic resistant Gram negatives were isolated in July—September 2007 ffrom cold dishes and food contact surfaces and utensils. Bacterial strains were submitted to susceptibility test and subtyped by random amplification of polymorphic DNA (RAPD). Forty-six of 55 (83.6%) food samples and 14 of 17 (82.3%) environmental swabs were culture positive for Gram negative bacilli resistant to at least one group of antibacterial drugs. A total of 134 antibiotic resistant strains, 51 fermenters and 83 non-fermenters, were recovered. Fermenters and non-fermenters showed frequencies as high as 97.8% of resistance to two or more groups of antibiotics and non fermenters were 28.9% resistant to more than three groups. Molecular typing detected 34 different profiles among the fermenters and 68 among the non-fermenters. Antibiotic resistance was very common among both fermenters and non-fermenters. However, the wide heterogeneity of RAPD patterns seems to support a prominent role of cross-contamination rather than a clonal expansion of a few resistant isolates. A contribution of commensal Gram negatives colonizing foods to a common bacterial resistance pool should not been overlooked.

## 1. Introduction

Resistance to various antibacterial drugs is rapidly emerging and posing a major challenge to Public Health. A comprehensive understanding of the most important dissemination routes of antimicrobial resistant bacterial (ARB) strains and resistance encoding genetic sequences is crucial to effectively control and minimize the problem [1]. 

Food appears to be an effective source for the acquisition by humans of drug resistant bacteria and drug resistance genes, but the extension and the actual consequences of this exposure are still insufficiently investigated [2–5]. Moreover, while occurrence and evolution of a foodborne resistant pathogen's incursion into various community and health-care associated settings has been frequently experienced and thoroughly studied, horizontal gene transfer events taking place between commensals and pathogens and between food-derived commensals and human commensals are to date poorly known [4–11]. 

Such transfers will likely be most successful when the host is simultaneously submitted to a selective pressure by an antimicrobial substance to which the involved organisms are resistant [1]. From this point of view, health-care associated settings are the environments where ARB and their resistance determinants are most likely to be present, a genetic horizontal transmission has more chances to occur, and the consequences in terms of therapeutic failures and costs might be more severe [1, 12, 13]. 

Hospital food service systems are considered one of the most critical segment of hospitality industry, where the client base is often a vulnerable group, and a strict and systematic monitoring of foodborne hazards has to be consistently applied [14]. 

This study aimed at detecting the presence of multidrug resistant Gram-negatives (MDR-GN) in food samples delivered through a plated service at the University general hospital “Azienda Ospedaliero-Universitaria Policlinico” (AOUP), Palermo, Italy. Food contact surfaces of equipment and utensils at the Hospital caterer food premise, where food was prepared, portioned and placed into the personalized trays were also sampled during an inspective visit. Patterns of antibacterial drug susceptibility and genetic heterogeneity of the MDR-GN were assessed.

## 2. Materials and Methods

### 2.1. Setting

This investigation was conducted at the University general hospital “Azienda Ospedaliero-Universitaria Policlinico” (AOUP), Palermo, Italy, in the period July–September 2007. At this hospital, the food service was contracted out to an external caterer who was employing a traditional cook and serve production scheme and a plated meal distribution system. In particular, food was being ordered according to the patient's choice up to 24 hours in advance. At the caterer plant, meals were prepared, assembled, and then plated using a conveyor belt with food handlers standing either side and serving appropriate portions into plates. Hot dishes were then placed into trays that were being in turn stacked into preheated cabinets, with cold dishes being placed into separate compartments, before transport and delivery to the hospital wards.

### 2.2. Food Samples

For the purpose of the study, cold dishes only that had not been submitted to thermal treatment were selected. Samples of 50 g approximately were daily collected at receipt in the hospital wards from lunch meals, by taking them from a preordered tray similar to that of a patient. Samples were immediately placed in a refrigerated container and kept at 4°C until transferred to the laboratory for testing (approximately 15 minutes). When delivered to the laboratory, 10 g of each food sample were aseptically weighted into a sterile Stomacher bag, and 90 mL of peptone water were then added. Samples were homogenised for 60 seconds and incubated for 24 hours at 37°C.

### 2.3. Food Contact Surfaces and Utensils

The inspection and the environmental sampling were carried out on September 2007. For food contact surfaces and utensils of the caterer premise, a swab sampling technique was used. The tip of each sterile cotton swab was moistened with sterile saline, pH 7.0, and then rolled repeatedly over each 10 cmq surface area. After the sampling, the swabs were placed aseptically into 10 mL of peptone water and transferred to the laboratory at chilled temperature. After the swabs were delivered to the laboratory, each tube containing the swab was vortexed 10 seconds to assure mixture of the sample and then incubated for 24 hours at 37°C.

### 2.4. Detection Method of Antibiotic-Resistant Gram Negatives

One MacConkey agar plate was inoculated with 0.2 mL of the 24-hour enrichment culture to obtain a continuous lawn after overnight incubation in ambient air at 37°C. Four antibiotic disks, containing amoxicillin-clavulanic acid (20/10 *μ*g), ceftazidime (30 *μ*g), gentamicin (10 *μ*g), and nalidixic acid (30 *μ*g), were placed on each plate before incubation, as previously described [15].

After incubation, plates were examined, and all colonies of different morphology growing into each antibiotic inhibition halo were Gram stained and subcultured for purity. All isolates were submitted to a biochemical screening by testing for oxidase and catalase activity and glucose fermentation and classified as Gram negative fermenters or non-fermenters. A complete biochemical identification by the system API 20E or API 20NE (bioMérieux, Marcy-l’Etoile, France) was deserved to some clustered isolates and to two extended-spectrum *β*-lactamase-(ESBL-) producing Gram negative fermenters. Biochemical characterization to the species level of the remaining isolates was thought to be unable to provide useful additional information, because of the largely unreliable results that are generally obtained from food isolates by the commercial phenotypic systems, including the API system, according to recent findings [16].

### 2.5. Antibiotic Susceptibility Testing

Susceptibility of each isolate to a panel of nine antimicrobial substances was assessed by disk diffusion on Mueller-Hinton agar plates, according to the Clinical and Laboratory Standards Institute (CLSI) guidelines [17]. The following antimicrobials were tested: amoxicillin-clavulanic acid, Amc (20/10 *μ*g), cefotaxime, Ctx (30 *μ*g), ceftazidime, Caz (30 *μ*g), ceftriaxone, Cro (30 *μ*g), gentamicin, Cn (10 *μ*g), netilmycin, Net (30 *μ*g), nalidixic acid, Na (30 *μ*g), ciprofloxacin, Cip (5 *μ*g), and tetracycline, Te (30 *μ*g). 

ESBL production was detected by decreased susceptibility or resistance to third-generation cephalosporins and the synergy between disks containing cefotaxime, ceftazidime, cefepime, and aztreonam and a disk containing amoxicillin-clavulanic acid [18].

For the purpose of the study, a Gram negative organism resistant to at least two different groups of antimicrobial agents (amoxicillin-clavulanic acid, cephalosporins, aminoglycosides, quinolones, tetracycline) was defined as MDR-GN.

### 2.6. Genotypic Analysis by Random Amplification of Polymorphic DNA (RAPD)

Single colonies growing on solid media were removed with a sterile plastic tip and resuspended in 100 *μ*L of sterile deionized water in a microcentrifuge tube. DNA extracts were prepared by boiling the suspensions for 10 minutes. After a quick spin to pellet the cell debris, the supernatants were used as DNA templates for subsequent amplifications or stored at −20°C until PCR was applied.

RAPD was performed as previously described [19, 20]. The PCR mixture contained 200 *μ*M each dNTP, 150 ng primer, buffer (10 mM Tris-HCl, pH 8.8; 1.5 mM MgCl_2_; 50 mM KCl; 0.1% Triton X-100), 2 U *Taq* DNA polymerase (Promega, Madison, WI, US), and 1 *μ*L whole-cell DNA in a total volume of 50 *μ*L. The amplification protocol consisted of the following steps: initial denaturation at 94°C for 5 minutes, followed by 40 cycles of denaturation (1 minute at 94°C), annealing (1 minute at 36°C) and extension (72°C for 2 minutes), and a final extension step (72°C for 10 minutes). 

The primers used in this study were ERIC2 (5′-ATGTAAGCTCCTGGGGATTCAC-3′) and M13 (5′-GAGGGTGGCGGTTCT-3′). Primer ERIC2 was primarily used, whereas M13 was used for confirmation of clustering .

Amplified PCR products were separated using 2% agarose gels, visualized by UV transillumination and photographed. A 1 Kbp or a 100 bp DNA ladder (Promega) was used as molecular weight. DNA fingerprints were compared by visual inspection and considered unique when they differed by at least one band, irrespectively of band intensity.

### 2.7. Statistical Analysis

Data were analyzed by the EpiInfo software (version 6.0, CDC, Atlanta, GA, US). Frequency analysis was performed with the chi-square test. Cross tabulation and chi-square or Fisher exact tests were performed to determine the relationship between resistance and some characteristics of isolates and food products processed for isolation of resistant Gram negatives. For some statistical analysis, susceptibility to antibiotics was categorized as a dichotomous variable by interpreting intermediate susceptibility as resistance. In all analyses, differences were considered statistically significant at *P* ≤ .05.

## 3. Results

### 3.1. Detection of ARB Food and Environmental Isolates

During the period July–September 2007, 55 food samples and 17 swabs from food contact surfaces and utensils were examined. Forty-six of 55 (83.6%) food samples and 14 of 17 (82.3%) environmental swabs proved to be culture positive for Gram negative bacilli resistant to at least one group of antibacterial drugs. 

A total of 115 different isolates of Gram negative bacilli resistant to at least one antimicrobial group were identified from the following food products: 22 from 18 samples of soft cheese, three from three samples of sliced ham, 20 from 12 samples of mixed ham and cheese dishes, and 70 from 24 samples of vegetables. 

The 19 resistant environmental isolates were, respectively, six from countertops, a centrifuge, a sink, and a conveyor belt in the washing area of fresh vegetables, nine from cutting boards, trays, and knives in the vegetables processing area, and four from a slicing machine and food contact surfaces in the cured meat working area.

### 3.2. Characterization of ARB Isolates

The 134 Gram negative isolates were biochemically categorized as 51 fermenters and 83 non-fermenters. Prevalence of resistant Gram negative fermenters versus non-fermenters did not significantly differed in the four groups of food products examined (*P* = .74).

**Figure 1:**
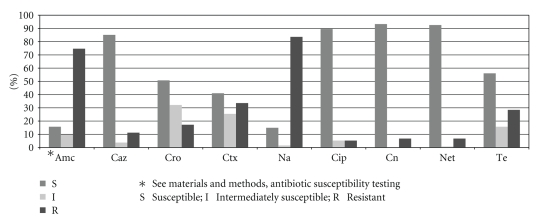
Prevalence of resistance, intermediate susceptibility, and susceptibility towards the antibacterial drugs tested among 134 Gram negative bacterial strains isolated from foods, food contact surfaces, and utensils.

Prevalence of the resistant, intermediate, or susceptible phenotype towards the antibacterial drugs tested among all isolates is illustrated in Figure 1. Resistances to nalidixic acid (83.6%) and amoxicillin-clavulanate (74.6%) were very frequent, followed by resistances to cefotaxime (33.6%) and tetracycline (28.4%). Statistically significant differences were detected between fermenters and nonfermenters in the prevalence of resistance to all antibacterial drugs, but two—nalidixic acid and gentamicin (Table 1). Moreover, fermenters were significantly more likely to exhibit resistance to ciprofloxacin and tetracycline, whereas non-fermenters to amoxicillin-clavulanate, cephalosporins, and netilmycin (Table 1).

A total of 29 different resistance patterns, 12 among fermenters and 20 among non-fermenters, respectively, were identified. The patterns and their distribution are summarized in Table 2.

**Figure 2:**
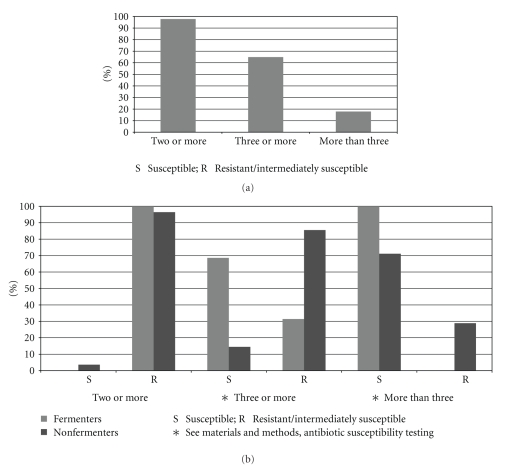
(a) Percent distribution among the 134 resistant Gram negative bacterial strains of resistance (including intermediate susceptibility) to at least two, at least three, and more than three antibacterial drugs. (b) Comparison between frequency of resistance to at least two, at least three, and more than three antibacterial drugs in Gram negative fermenters and nonfermenters.

Figures 2(a)  and 2(b)  show the prevalence of resistance to at least two, at least three and more than three groups of antibacterial drugs among all isolates and after stratifying fermenters and non-fermenters. Non-fermenters were significantly associated (*P* < .01) with a higher percentage of resistance to three or more than three groups of antibiotics.

Two fermentative isolates that proved to be ESBL producing by the modified double-disk synergy test were identified as *Enterobacter cloacae*.

### 3.3. Molecular Typing and Clustering of ARB Isolates

**Figure 3:**
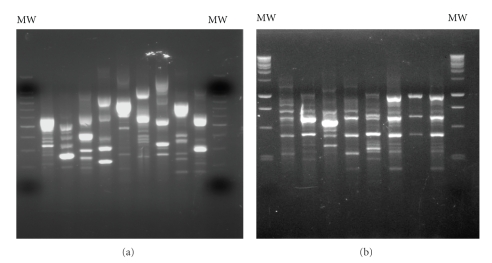
ERIC-2 RAPD patterns of representative Gram negative fermenters (a) and non-fermenters (b). MW = molecular weight. (a) 100 bp; (b) 1 Kbp.

Molecular typing of the resistant Gram negative bacilli by RAPD with the ERIC2 primer detected 34 different profiles among the 51 fermenters and 68 among the 83 non-fermenters (Figures 3(a)  and 3(b)). Eight RAPD patterns among fermenters and nine among non-fermenters included between two and ten isolates. However, 15 of 17 clusters contained two or three isolates, whereas only two RAPD patterns—F26 and NF28, respectively—were attributed to ten fermenting and eight non-fermenting Gram negative isolates. Isolates sharing F26 and NF28 RAPD profiles were biochemically identified, respectively, as *Klebsiella oxytoca* and *Pseudomonas aeruginosa*. 

All clusters were confirmed by using the M13 primer. The two ESBL positive *E. cloacae* showed unique RAPD profiles.

Clustered isolates were significantly (*P* = .02) more likely to be resistant to three antibiotic groups and less likely (*P* = .01) to be resistant to more than three antibiotic groups. No association was found between clustering and resistance to at least two antibiotic groups (*P* = .25). Among the 10 *K. oxytoca* isolates with RAPD pattern F26, eight were resistant to Na, Cip, and Te and two to Na and Te only. The eight *P. aeruginosa* with pattern NF28 had more heterogeneous resistance patterns: three were Amc Cro Ctx Na, two Amc Cro Ctz Na Te, and one each Amc Caz Cro Ctx Na, and Amc Ctx Na, Amc Na Te, respectively.

Clustering was not significantly associated to any food product (*P* = .43). 

Except for the two larger ones, the remaining clusters included isolates recovered from food samples in an interval of time ranging from 0, when their isolation was made from different food products sampled in the same date, to 10 days. The 10 isolates of *K. oxytoca* with RAPD pattern F26 had been recovered from seven leaf lettuce salads and three dishes containing mixed ham and soft cheese distributed in different days. The seven *P. aeruginosa* isolates RAPD pattern NF28 were also sampled in different days: they were, respectively, five from leaf green salads, one from a tomato salad, and one from a soft cheese based dish. The interval of time between the first and the last isolation was in both case of 50 days.

## 4. Discussion

The role of food within the overall framework of human exposure to drug resistant bacteria has been until now insufficiently investigated from a Public Health perspective. Many studies have focused on the contribute of antimicrobial resistance to the severity of the hazard posed by foodborne pathogens and the specific measures to be adopted along the food chain to minimize the current trend of some pathogenic bacteria, such as *Salmonella*, towards acquiring resistance to fluoroquinolone and 3rd and 4th generation cephalosporins [21–23]. However, the issue of commensal bacteria as a potential hazard both directly as opportunistic organisms and indirectly as carriers of resistance genes has been only partially explored to date [11, 24]. 

To contribute additional information about food-mediated exposure to Gram negative ARB, we investigated the occurrence of these organisms in food products processed in a catering premise and delivered to hospitalized patients. Indeed, a considerable portion of these subjects belongs to a population subgroup of consumers where colonization by resistant bacteria and selective pressure due to use of antibacterial drugs may interact within a supportive environment and generate more severe health risks [12, 13]. 

A proportion as high as 83.6% of food samples tested positive for Gram negative resistant to one or more groups of antibiotics, with a great heterogeneity of resistance patterns and RAPD patterns among both fermenters and nonfermenters. Moreover, both Gram negative groups showed frequencies as high as 97.8% of resistance to two or more groups of antibiotics. Nonfermenters, in particular, were 28.9% resistant to more than three groups. Markedly lower resistance prevalences have been previously described in *Enterobacteriaceae* isolated from minced meat and vegetables, but with a less sensitive detection method lacking of the preliminary step on a selective culture medium [25, 26]. Hence, a comparison would be inherently biased due to the preselection of our strain set on the basis of the resistance to at least one antibiotic. Moreover, in a paper by Bezanson et al. [8], describing resistance to ten antibiotics in oxidase-positive bacteria from raw salad vegetables, resistance to some antibiotics, such as nalidixic acid or aminoglycosides, in Gram negatives was less frequent than our findings. Again methodological differences, mainly in the antibiotic susceptibility testing, and the inclusion of an enrichment step in our study could contribute to explain such inconsistency. An important additional role could be supposedly attributed to our choice to examine processed foods, since a qualitative/quantitative change of their commensal flora could have been occurring through the subsequent stages of food chain in the catering premise. It is unknown, on the other hand, whether antimicrobial resistant bacteria may have a selective advantage upon the susceptible ones in surviving or multiply in a food processing plant, except for the reported association of resistances to antibiotics and biocides [27]. 

Of particular concern appears the high prevalence of resistance to nalidixic acid and, to a less extent, cefotaxime. Literature suggests that resistance to nalidixic acid determined by the disk diffusion method may be a reliable indicator of decreased susceptibility to ciprofloxacin [28]. Resistance to this last antibiotic in our data is significantly more frequent among fermentative Gram negatives, that include *Enterobacteriaceae*, a family where fluoroquinolone resistance is emerging in opportunistic and pathogen members in both hospital and community settings [3, 21, 22, 27]. Spreading of *β*-lactamases producing bacteria and codifying sequences is a further worrying feature in the complex epidemiology of drug resistance [5]. Detection of a high proportion of cephalosporin-resistant isolates along with the isolation of two ESBL-producing *E. cloacae* isolates confirms previous results by other Authors [5].

A further finding that deserves consideration is the large heterogeneity of RAPD patterns, that excludes clonal expansion as a possible reason of the high prevalence of antibiotic resistances in foods. Moreover, identification of some clusters of fermenter and non-fermenter isolates in intervals of time ranging between 0 and 50 days proves the persistence in the food processing environment of resistant organisms and, consequently, the potential effectiveness of Good Hygienic Practices in minimizing their diffusion. 

Our study has some limits. Firstly, sampling has been carried out in a single food catering premise. Consequently, the results could have been heavily influenced by the hygienic conditions of the plant, and their generalizability could be questionable. Furthermore, the issue of location and horizontal transferability of resistance genetic determinants have not been addressed. Intrinsic resistances to some antibiotics, for example, presence of AmpC mediated *β*-lactames or tetracycline resistance in various *Enterobacteriaceae,* could have likely overestimated the multiresistance prevalence. Finally, no evidence has been searched for of a possible relationship with colonizing isolates among patients staying in the hospital during the study.

## 5. Conclusions

Based on our results, a contribution of commensal ARB Gram negatives colonizing processed foods consumed without further thermal treatment or processing to a common resistance pool should not been overlooked. This is a disturbing finding when considering the possible impact of a daily administration of resistant bacteria to a susceptible population, particularly those with defective immune systems or comorbidities and those receiving antibiotic treatment. Understanding the routes connecting the resistant bacteria and genetic resistance determinants to humans, including the role of food vehicles, is critical to define effective strategies to control this problem. The consistent and effective application of good food hygiene practices is a key issue in the prevention and control of food contamination with antimicrobial-resistant pathogenic and commensal bacteria.

## Figures and Tables

**Table 1:** Frequency of resistance, intermediate susceptibility, and susceptibility to the antibacterial drugs tested in fermenters and nonfermenters.

	Amc*§		Caz*		Cro*		Ctx*		Na		Cip*		Cn		Net*		Te*	
	F	NF	F	NF	F	NF	F	NF	F	NF	F	NF	F	NF	F	NF	F	NF
S (%)	29.4	7.2	96.1	78.3	94.1	20.0	94.1	8.4	0	15.7	74.5	98.8	96.1	91.6	100	88.0	37.3	67.5
I (%)	17.7	4.8	0.0	6.0	2.0	50.6	0.0	41.0	13.7	2.4	13.7	0.0	0.0	0.0	0.0	1.2	3.9	22.9
R (%)	52.9	88.0	3.9	15.7	3.9	25.3	5.9	50.6	86.3	81.9	11.8	1.2	3.9	8.4	0.0	10.8	58.8	9.6

*P < .05 § See Materials and Methods, Antibiotic susceptibility testing. S = susceptible; I = intermediately susceptible; R = resistant. F = fermenters; NF = non-fermenters.

**Table 2:** Resistance patterns to antibacterial drugs tested in fermenters and nonfermenters.

Fermenters			Non-fermenters	
Resistance pattern*	Number of isolates		Resistance pattern*	Number of isolates
				
Amc Na	18		Amc Cro Ctx Na	19
Amc Na Te	9		Amc Cro Ctx Na Te	17
Na Cip Te	8		Amc Ctx Na	11
Amc Te	4		Amc Caz Cro Ctx Na	9
Na Te	3		Amc Cro Ctx Cn Net Te	4
Amc Na Cip Te	2		Amc Na	4
Na Cn	2		Caz Cro Ctx	3
Amc	1		Amc Cro Ctx	2
Amc Caz Cro Ctx	1		Amc Cro Ctx Na	2
Amc Caz Cro Ctx Te	1		Amc Ctx Na Te	2
Cro Ctx Na Te	1		Amc Caz Cro Ctx Cn Net	1
Na Cip	1		Amc Caz Cro Ctx Na Net Te	1
			Amc Caz Cro Ctx Net	1
			Amc Cro Ctx Te	1
			Amc Cro Na	1
			Amc Na Net	1
			Amc Na Te	1
			Caz Cn Net	1
			Caz Ctx	1
			Cro Ctx Na Te	1

*See Materials and Methods, Antibiotic susceptibility testing.
